# Region- or state-related differences in expression and activation of extracellular signal-regulated kinases (ERKs) in naïve and pain-experiencing rats

**DOI:** 10.1186/1471-2202-8-53

**Published:** 2007-07-24

**Authors:** She-Wei Guo, Ming-Gang Liu, Ya-Li Long, Li-Ying Ren, Zhuo-Min Lu, Hou-You Yu, Jun-Feng Hou, Hua Li, Cui-Ying Gao, Xiu-Yu Cui, Yang-Yuan An, Junfa Li, Lan-Feng Zhao, Jun Chen

**Affiliations:** 1 Institute for Biomedical Sciences of Pain, Capital Medical University, Beijing 100069, P. R. China; 2 Institute for Biomedical Sciences of Pain and Institute for Functional Brain Disorders, Tangdu Hospital, Fourth Military Medical University, Xi'an 710038, P. R. China

## Abstract

**Background:**

Extracellular signal-regulated kinase (ERK), one member of the mitogen-activated protein kinase (MAPK) family, has been suggested to regulate a diverse array of cellular functions, including cell growth, differentiation, survival, as well as neuronal plasticity. Recent evidence indicates a role for ERKs in nociceptive processing in both dorsal root ganglion  and spinal cord. However, little literature has been reported to examine the differential distribution and activation of ERK isoforms, ERK1 and ERK2, at different levels of pain-related pathways under both normal and pain states. In the present study, quantitative blot immunolabeling technique was used to determine the spatial and temporal expression of ERK1 and ERK2, as well as their activated forms, in the spinal cord, primary somatosensory cortex (SI area of cortex), and hippocampus under normal, transient pain and persistent pain states.

**Results:**

In naïve rats, we detected regional differences in total expression of ERK1 and ERK2 across different areas. In the spinal cord, ERK1 was expressed more abundantly than ERK2, while in the SI area of cortex and hippocampus, there was a larger amount of ERK2 than ERK1. Moreover, phosphorylated ERK2 (pERK2), not phosphorylated ERK1 (pERK1), was normally expressed with a high level in the SI area and hippocampus, but both pERK1 and pERK2 were barely detectable in normal spinal cord. Intraplantar saline or bee venom injection, mimicking transient or persistent pain respectively, can equally initiate an intense and long-lasting activation of ERKs in all three areas examined. However, isoform-dependent differences existed among these areas, that is, pERK2 exhibited stronger response than pERK1 in the spinal cord, whereas ERK1 was more remarkably activated than ERK2 in the S1 area and hippocampus.

**Conclusion:**

Taken these results together, we conclude that: (1) under normal state, while ERK immunoreactivity is broadly distributed in the rat central nervous system  in general, the relative abundance of ERK1 and ERK2 differs greatly among specific regions; (2) under pain state, either ERK1 or ERK2 can be effectively phosphorylated with a long-term duration by both transient and persistent pain, but their response patterns differ from each other across distinct regions; (3) The long-lasting ERKs activation induced by bee venom injection is highly correlated with our previous behavioral, electrophysiological, morphological and pharmacological observations, lending further support to the functional importance of ERKs-mediated signaling pathways in the processing of negative consequences of pain associated with sensory, emotional and cognitive dimensions.

## Background

It has been well known that nociceptive information is transmitted along multiple ascending systems to the brain [1-3]. Consequently, a diffuse network of brain centers, each of which contributes to sensory, emotional and cognitive dimensions of pain [4,5], will be activated during the complex experience of pain [1,6-8]. Among these brain regions, primary somatosensory cortex (SI area of cortex), with its anatomical interconnections to nociceptive signal-carrying pathways, has been proposed to be potently activated following noxious stimulation and to subserve the somatosensory-discriminative aspects of pain, such as location and intensity coding of pain [6-10]. In addition, the hippocampus, an integral component of the 'limbic' system [11,12], may also contribute to the negative affect and evaluation/cognition of pain experience [13,14]. It is speculated that nociceptive input may be integrated in the hippocampus with the context memory to allow a full appreciation of the meanings and dangers of extraneous pain-producing stimuli. However, so far, very little is known about the precise cellular and molecular mechanisms underlying the pain processing in both SI area and hippocampus.

The generic term of mitogen-activated protein kinases (MAPKs) is used to denote a family of signal transduction molecules that transduce a broad range of extracellular stimuli into diverse intracellular responses by producing changes in transcriptional modulations of key genes as well as posttranslational modifications of target proteins [15-17]. The ERK (extracellular signal-regulated kinase) members of the MAPK family are originally identified as the primary effectors of growth factor receptor signaling and supposed to be involved in the cellular proliferation, differentiation and survival processes [18-20]. Nevertheless, recent evidence suggests a role for ERKs in regulating neuronal plasticity, such as long-term synaptic potentiation (LTP), long-term depression (LTD) that underlie learning and memory functions [21-24]. Activated forms of ERKs act both in the peripheral nociceptor terminal and the dorsal horn to produce pain hypersensitivity within an early and short period of their activation by nociceptor afferent input evoked by acute noxious stimulus, an effect that is likely due to posttranslational processing [25-27]. Further, peripheral inflammation and nerve injury can also induce sustained activation of ERKs in both dorsal root ganglion (DRG) and the second order dorsal horn neurons (or glial cells), which then lead to enhanced gene expression and alterations in the neuronal phenotypes, thus contributing to both inflammatory and neuropathic pain [28-32]. In spite of these results, few study has been conducted to determine the possible relationship between ERKs activation in higher brain regions, such as SI area and hippocampus, as described above, and multidimensions of pain.

There has been increasing experimental evidence showing that the clinical pathological pain, charicterized by persistent pain and hyperalgesia, differs greatly from physiological pain, which is acute and transient, warning of potential or actual tissure or nerve damage [33-36]. Therefore, the aim of the present series of experiments is to assess the spatial- and temporal-related changes in phosphorylation (activation) and protein expression of ERKs, mainly ERK1 and ERK2, in the spinal cord dorsal horn, SI area and hippocampus under both physiological pain (transient pain) and pathological pain (persistent pain) states. To attain this goal, we adopted two well-charecterized animal models: (1) subcutaneous (s.c.) injection of 0.9% isotonic saline solution in conscious rats as the physilogical (transient) pain model, for the injury produced by the injection itself can be perceived as acute pain sensation by animals [29]; (2) s.c. injection of a solution containing the whole bee venom in rats as the pathological (persistent) pain model [33,37]. Our previous behavioral studies have demonstrated that s.c. injection of bee venom into the plantar surface of one hindpaw in conscious rats could produce persistent spontaneous nociception (PSN), heat or mechanical hyperalgesia and also peripheral inflammation [33,38-40]. Furthermore, our electrophysiological experiments suggest that the bee venom model possesses many advantages over the formalin test, another persistent pain model, and may be more appropriate in evaluation of the mechanisms underlying clinical pathological pain [41-43]. Besides, we also try to provide an initial investigation into the differential regional distribution of ERK isoforms, including their activated forms, across different areas under normal state, in hopes of getting a new insight into their isoform-specific and region-dependent charecteristics in normal expression. Here, we reported region- and state-related differences in phosphorylation and expression of ERK isoforms in the rat central nervous system (CNS).

## Results

### Effects of s.c. injection of saline or bee venom on the phosphorylation of ERK1 and ERK2 in the spinal cord

**Figure 1:**
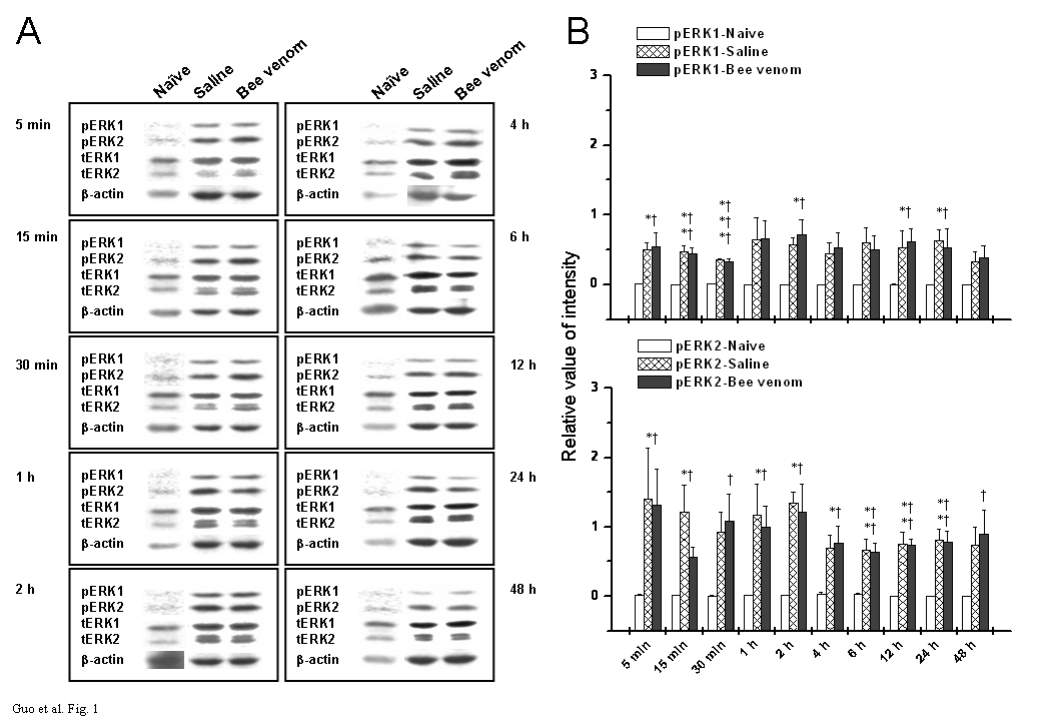
Temporal changes in phosphorylation and expression of ERKs in ipsilateral spinal cord dorsal horn under three different states. (A) Representative Western blots of spinal cord homogenates from three groups of rats at indicated time points. Left panel in each rectangle represents immunodetection of phosphorylated-ERK1/2 (pERK1/2), total ERK1/2 (tERK1/2), and beta-actin, which is included as loading control. Right panel in each rectangle shows raw bands corresponding to the left panel. (B) Densitometric quantification from (A) is shown. The pERKs bands were densitized and normalized to tERKs immunoblotted from the same membrane to demonstrate the phosphorylation levels of ERKs. Upper panel of B denotes the time course of ERK1 activation under three assigned states, while lower panel of B illustrates the time-related changes in activation of ERK2. Points represent the mean ± S.E.M. from three separate experiments. * *P *< 0.05, ** *P *< 0.01, *** *P *< 0.001, saline-treated rats versus naïve control; † *P *< 0.05, †† *P *< 0.01, ††† *P *< 0.001, bee venom-treated rats versus naïve control.

To test the differential expression patterns of ERK1 and ERK2, as well as their activated forms, in the spinal cord under normal, transient pain and persistent pain states, different groups of rats (n = 3/group at each time point) were injected intraplantarly with 50 μl 0.9% sterile saline or 50 μl whole bee venom or without any treatment and homogenates of the spinal cord tissue obtained at various time points (as described in Materials and methods) were subsequently probed for phospho-ERK1/2 (pERK1/2) as well as total ERK1/2 (tERK1/2) using one kind of primary antibody that could detect these two bands (44 kDa for ERK1; 42 kDa for ERK2) on the same membrane simultaneously. The representative original immunoblotting bands detected in ipsilateral spinal cord dorsal horn obtained from three groups of rats were shown in Fig. 1A. In the normal spinal cord of naïve rats, both pERK1 and pERK2 were barely detectable at whatever time point we examined, although there was a considerable amount of total ERKs constitutively expressed, with ERK1 being more abundant than ERK2. However, s.c. administration of bee venom into the plantar surface of one hindpaw, which could produce a prolonged tonic, monophasic nociceptive response characterized by continuously flinching or lifting the injected paw for 1–2 h [37,38], significantly elevated the phosphorylation level of both ERK1 and ERK2 in the ipsilateral spinal cord (Fig. 1A, Tables 1 and 2). Interestingly, saline-treated rats, which exhibited typical behavioral manifestation of acute and transient pain during the process of injection, also displayed a higher level of both pERK1 and pERK2 compared with control (Fig. 1A, Tables 1 and 2). Fig. 1B illustrates quantitative analysis of the data shown in Fig. 1A. This column graph clearly demonstrated that both saline-induced transient pain and bee venom-induced persistent pain can equally elicit the enhanced expression of activated ERK1 or ERK2 in the ipsilateral side of the spinal cord dorsal horn for a long period (about 24–48 h). There were not any significant differences detected between saline- and bee venom-treated rats in terms of ERK1 or ERK2 activation (Fig. 1). In spite of the great differences in basal expression amounts of total protein between ERK1 and ERK2 in the normal spinal cord, no significant alterations in tERK1 or tERK2 level were detected following noxious stimulation, transient or persistent, when compared to naïve rats, respectively (data not shown).

**Table 1:** Region- or state-related differences in activation of ERK1 in naïve and pain-experiencing rats.

	Spinal cord dorsal horn			S1 area of cortex			Hippocampus		
	
	Naïve	Saline	Bee venom	Naïve	Saline	Bee venom	Naïve	Saline	Bee venom
5 min									
Ipsil	2.61 × 10^-3 ^± 2.21 × 10^-3^	0.50 ± 0.11*	0.54 ± 0.20†	2.80 × 10^-3 ^± 2.80 × 10^-3^	2.56 ± 1.83	1.08 ± 0.38	0.04 ± 0.03	0.58 ± 0.33	0.93 ± 0.44
Contl	0.02 ± 0.01	0.57 ± 0.31	0.52 ± 0.32	3.32 × 10^-3 ^± 3.32 × 10^-3^	0.84 ± 0.30*	0.61 ± 0.24†	0.17 ± 0.13	0.87 ± 0.31	0.81 ± 0.28
15 min									
Ipsil	8.49 × 10^-4 ^± 5.49 × 10^-4^	0.47 ± 0.08**	0.44 ± 0.09††	1.63 × 10^-3 ^± 1.63 × 10^-3^	0.93 ± 0.30*	0.49 ± 0.23†	0.02 ± 0.01	0.60 ± 0.42	0.55 ± 0.30
Contl	0.02 ± 0.02	0.34 ± 0.12**	0.30 ± 0.07††	2.61 × 10^-4 ^± 2.61 × 10^-4^	0.63 ± 0.28*	0.95 ± 0.40†	0.07 ± 0.04	0.40 ± 0.08*	0.32 ± 0.08†
30 min									
Ipsil	2.50 × 10^-3 ^± 1.98 × 10^-3^	0.36 ± 0.01***	0.32 ± 0.05†††	1.14 × 10^-3 ^± 1.14 × 10^-3^	0.52 ± 0.16*	0.51 ± 0.20†	4.16 × 10^-3 ^± 3.43 × 10^-3^	0.50 ± 0.19*	0.72 ± 0.10†
Contl	0.01 ± 0.01	0.27 ± 0.10	0.25 ± 0.08	4.93 × 10^-4 ^± 4.93 × 10^-4^	0.95 ± 0.16*	1.48 ± 0.58†	0.03 ± 0.01	0.38 ± 0.13*	0.75 ± 0.13†
1 h									
Ipsil	8.45 × 10^-4 ^± 7.28 × 10^-4^	0.64 ± 0.32	0.67 ± 0.26	0	0.65 ± 0.34	0.29 ± 0.07	3.95 × 10^-3 ^± 3.40 × 10^-3^	0.64 ± 0.50	0.58 ± 0.42
Contl	7.89 × 10^-3 ^± 6.95 × 10^-3^	0.36 ± 0.04**	0.29 ± 0.06††	0	0.48 ± 0.28**	0.52 ± 0.22††	0.02 ± 0.02	0.62 ± 0.41	2.23 ± 1.79
2 h									
Ipsil	4.71 × 10^-4 ^± 4.48 × 10^-4^	0.57 ± 0.10*	0.71 ± 0.22†	2.61 × 10^-4 ^± 2.61 × 10^-4^	1.07 ± 0.68	0.56 ± 0.40	0.02 ± 0.02	0.49 ± 0.22	0.66 ± 0.28
Contl	5.99 × 10^-3 ^± 4.26 × 10^-3^	0.27 ± 0.07	0.38 ± 0.15†	0	0.57 ± 0.13*	0.55 ± 0.06†	0.03 ± 0.03	0.52 ± 0.18	0.49 ± 0.28
4 h									
Ipsil	5.53 × 10^-4 ^± 3.33 × 10^-4^	0.44 ± 0.16	0.53 ± 0.21	0	0.65 ± 0.37	0.82 ± 0.52	0.05 ± 0.03	0.37 ± 0.08*	0.59 ± 0.12†
Contl	2.40 × 10^-3 ^± 1.43 × 10^-3^	0.35 ± 0.11*	0.34 ± 0.09†	0.07 ± 0.06	1.32 ± 0.48*	1.08 ± 0.16†	0.07 ± 0.05	0.33 ± 0.04	0.52 ± 0.25
6 h									
Ipsil	1.13 × 10^-3 ^± 5.75 × 10^-4^	0.60 ± 0.22	0.49 ± 0.21	6.79 × 10^-4 ^± 6.79 × 10^-4^	0.37 ± 0.09**	0.42 ± 0.03††	0.04 ± 0.02	1.38 ± 0.83	1.58 ± 0.68
Contl	6.00 × 10^-3 ^± 5.12 × 10^-3^	0.49 ± 0.06***	0.44 ± 0.03†††	0.08 ± 0.08	0.91 ± 0.53	0.60 ± 0.42	0.08 ± 0.06	1.25 ± 0.30*	0.93 ± 0.17†
12 h									
Ipsil	1.59 × 10^-3 ^± 8.16 × 10^-4^	0.53 ± 0.24*	0.62 ± 0.18†	0	0.53 ± 0.19**	0.54 ± 0.17††	7.94 × 10^-3 ^± 5.17 × 10^-3^	0.75 ± 0.09***	0.85 ± 0.16†††
Contl	1.49 × 10^-3 ^± 6.99 × 10^-4^	0.45 ± 0.05***	0.43 ± 0.06†††	4.17 × 10^-4 ^± 4.17 × 10^-4^	0.71 ± 0.46	1.02 ± 0.61	0.09 ± 0.09	1.16 ± 0.44*	1.28 ± 0.21†
24 h									
Ipsil	7.32 × 10^-4 ^± 4.13 × 10^-4^	0.63 ± 0.15*	0.53 ± 0.26†	0	0.62 ± 0.29	1.93 ± 1.52	1.97 × 10^-4 ^± 1.97 × 10^-4^	1.28 ± 0.70	0.87 ± 0.40
Contl	1.74 × 10^-3 ^± 8.96 × 10^-4^	0.39 ± 0.09**	0.35 ± 0.05††	5.15 × 10^-4 ^± 5.15 × 10^-4^	1.42 ± 0.52*	0.48 ± 0.05†	0.05 ± 0.05	2.02 ± 0.72	0.97 ± 0.22
48 h									
Ipsil	5.66 × 10^-4 ^± 3.37 × 10^-4^	0.33 ± 0.14	0.38 ± 0.18	1.84 × 10^-3 ^± 1.84 × 10^-3^	0.08 ± 0.04	0.16 ± 0.04†	7.45 × 10^-3 ^± 5.32 × 10^-3^	0.76 ± 0.02***	0.50 ± 0.04†††
Contl	6.04 × 10^-3 ^± 4.18 × 10^-3^	0.14 ± 0.02*	0.24 ± 0.05†††	6.85 × 10^-4 ^± 6.85 × 10^-4^	0.90 ± 0.83	0.88 ± 0.82	5.59 × 10^-3 ^± 5.32 × 10^-3^	0.65 ± 0.16*	0.36 ± 0.08†

Notes: Data are mean ± SEM. for the immunoblotted band intensities of phospho-ERK1 normalized to those of the loading control (total ERK1) obtained from three rats per group. Values from ipsilateral (Ipsil.) and contralateral (Contl.) sides of three indicated areas at ten time points are shown. * *P *< 0.05, ** *P *< 0.01, *** *P *< 0.001, saline-treated rats versus naïve control. † *P *< 0.05, †† *P *< 0.01, ††† *P *< 0.001, bee venom-treated rats versus naïve control.

**Table 2:** Region- or state-related differences in activation of ERK2 in naïve and pain-experiencing rats.

	Spinal cord dorsal horn			S1 area of cortex			Hippocampus		
	
	Naïve	Saline	Bee venom	Naïve	Saline	Bee venom	Naïve	Saline	Bee venom
5 min									
Ipsil	0.01 ± 0.01	1.40 ± 0.74*	1.32 ± 0.51†	0.43 ± 0.08	1.03 ± 0.31	0.85 ± 0.18	0.53 ± 0.18	0.61 ± 0.29	0.81 ± 0.13
Contl	0.02 ± 5.12 × 10^-3^	1.80 ± 0.36**	1.93 ± 0.36††	0.36 ± 0.05	0.86 ± 0.20	0.79 ± 0.18	0.58 ± 0.18	0.52 ± 0.23	0.77 ± 0.31
15 min									
Ipsil	9.51 × 10^-3 ^± 3.94 × 10^-3^	1.21 ± 0.40*	0.56 ± 0.15†	0.37 ± 0.10	0.91 ± 0.17	0.64 ± 0.22	0.55 ± 0.21	0.70 ± 0.10	0.67 ± 0.24
Contl	0.01 ± 7.74 × 10^-3^	1.50 ± 0.31**	1.31 ± 0.28††	0.37 ± 0.04	0.75 ± 0.20	0.79 ± 0.15	0.49 ± 0.14	0.62 ± 0.18	0.71 ± 0.16
30 min									
Ipsil	6.09 × 10^-3 ^± 1.20 × 10^-3^	0.93 ± 0.29	1.08 ± 0.40†	0.25 ± 0.10	0.72 ± 0.22	0.59 ± 0.20	0.49 ± 0.19	0.64 ± 0.27	0.79 ± 0.25
Contl	0.03 ± 0.02	1.45 ± 0.30*	1.34 ± 0.32†	0.26 ± 0.06	0.78 ± 0.22**	0.81 ± 0.12††	0.46 ± 0.17	0.52 ± 0.06	0.69 ± 0.07
1 h									
Ipsil	0.01 ± 7.56 × 10^-3^	1.16 ± 0.46*	1.00 ± 0.30†	0.16 ± 0.08	0.89 ± 0.45	0.91 ± 0.33	0.38 ± 0.17	1.04 ± 0.34	1.22 ± 0.27
Contl	0.06 ± 0.04	1.33 ± 0.20**	1.26 ± 0.20††	0.15 ± 0.07	0.67 ± 0.27	0.55 ± 0.22	0.44 ± 0.15	1.13 ± 0.40	5.29 ± 4.20
2 h									
Ipsil	9.31 × 10^-3 ^± 2.83 × 10^-3^	1.35 ± 0.16*	1.21 ± 0.41†	0.22 ± 0.11	0.62 ± 0.15*	0.52 ± 0.04†	0.47 ± 0.19	1.08 ± 0.12*	1.43 ± 0.17††
Contl	0.03 ± 0.02	1.21 ± 0.19*	1.52 ± 0.40††	0.22 ± 0.10	0.53 ± 0.12*	0.65 ± 0.11†	0.46 ± 0.17	1.19 ± 0.27	1.14 ± 0.31
4 h									
Ipsil	0.03 ± 0.03	0.69 ± 0.19*	0.76 ± 0.25†	0.25 ± 0.07	0.73 ± 0.17*	0.77 ± 0.14†	0.61 ± 0.31	0.94 ± 0.28	1.38 ± 0.13
Contl	0.02 ± 9.34 × 10^-3^	1.11 ± 0.12**	1.40 ± 0.33††	0.40 ± 0.05	0.93 ± 0.23*	1.10 ± 0.23†	0.55 ± 0.16	0.74 ± 0.05	1.00 ± 0.16
6 h									
Ipsil	0.03 ± 0.01	0.67 ± 0.16**	0.63 ± 0.14††	0.34 ± 0.08	1.19 ± 0.44	0.78 ± 0.13	0.75 ± 0.18	1.38 ± 0.36	1.52 ± 0.27
Contl	0.02 ± 6.72 × 10^-3^	1.20 ± 0.09***	1.19 ± 0.14†††	0.32 ± 0.05	1.34 ± 0.63	0.93 ± 0.18	0.67 ± 0.17	1.85 ± 0.57	1.49 ± 0.20
12 h									
Ipsil	3.31 × 10^-3 ^± 1.67 × 10^-3^	0.75 ± 0.18**	0.73 ± 0.09††	0.26 ± 0.04	0.82 ± 0.24	0.87 ± 0.20	0.55 ± 0.24	1.15 ± 0.27	1.27 ± 0.14
Contl	0.04 ± 5.41 × 10^-3^	1.38 ± 0.19**	1.58 ± 0.42††	0.19 ± 0.05	1.43 ± 0.63	1.64 ± 0.58	0.64 ± 0.17	2.11 ± 0.89	1.63 ± 0.14
24 h									
Ipsil	2.81 × 10^-3 ^± 7.82 × 10^-4^	0.81 ± 0.16**	0.79 ± 0.15††	0.11 ± 0.04	1.12 ± 0.59	2.07 ± 1.45	0.44 ± 0.17	1.13 ± 0.40	1.00 ± 0.28
Contl	0.01 ± 7.74 × 10^-3^	1.58 ± 0.34*	1.06 ± 0.15†	0.09 ± 0.03	1.75 ± 0.47	2.22 ± 1.74	0.61 ± 0.33	2.77 ± 0.93	1.24 ± 0.23
48 h									
Ipsil	3.65 × 10^-3 ^± 2.52 × 10^-3^	0.74 ± 0.26	0.90 ± 0.34†	0.18 ± 0.06	0.56 ± 0.23	0.48 ± 0.15	0.56 ± 0.27	1.22 ± 0.18	0.76 ± 0.08
Contl	0.08 ± 0.07	1.63 ± 0.21*	2.07 ± 0.66†	0.07 ± 0.01	0.65 ± 0.38	0.73 ± 0.36	0.70 ± 0.38	1.22 ± 0.09	0.86 ± 0.27

Notes: Data are mean ± SEM. for the immunoblotted band intensities of phospho-ERK2 normalized to those of the loading control (total ERK2) obtained from three rats per group. Values from ipsilateral (Ipsil.) and contralateral (Contl.) sides of three indicated areas at ten time points are shown. * *P *< 0.05, ** *P *< 0.01, *** *P *< 0.001, saline-treated rats versus naïve control. † *P *< 0.05, †† *P *< 0.01, ††† *P *< 0.001, bee venom-treated rats versus naïve control.

From the time course of ERK1 and ERK2 activation in response to peripheral painful stimulation (Fig. 1B), however, we could find remarkable differences between the response properties of ERK2 and ERK1 in terms of both immunoreactive intensity and duration, with the former being more susceptible and exhibiting stronger response than the latter. In fact, significant ERK2 activation was elicited immediately after injection, and maintained permanently until the end of the experiment. This new result of our experiment supports a piece of indirect evidence to the proposition that pERK2, but not pERK1, might behave as a more important intracellular signaling mediator in the spinal cord in response to peripheral noxious stimulation, although both activated enzymes were rarely presented in the spinal cord under normal, unstimulated state.

In the present study, we also assessed the state-dependent, time-related changes in phosphorylation of both forms of ERKs in contralateral side of the spinal cord with similar results obtained (Tables 1 and 2). These results indicate that ERKs, as important signaling intermediaries, are abundantly and broadly expressed in the spinal cord normally (Fig. 1A), enabling them to respond both rapidly and extensively when those cells receive environmental noxious stimuli.

### Effects of s.c. injection of saline or bee venom on the phosphorylation of ERK1 and ERK2 in the S1 area

**Figure 2:**
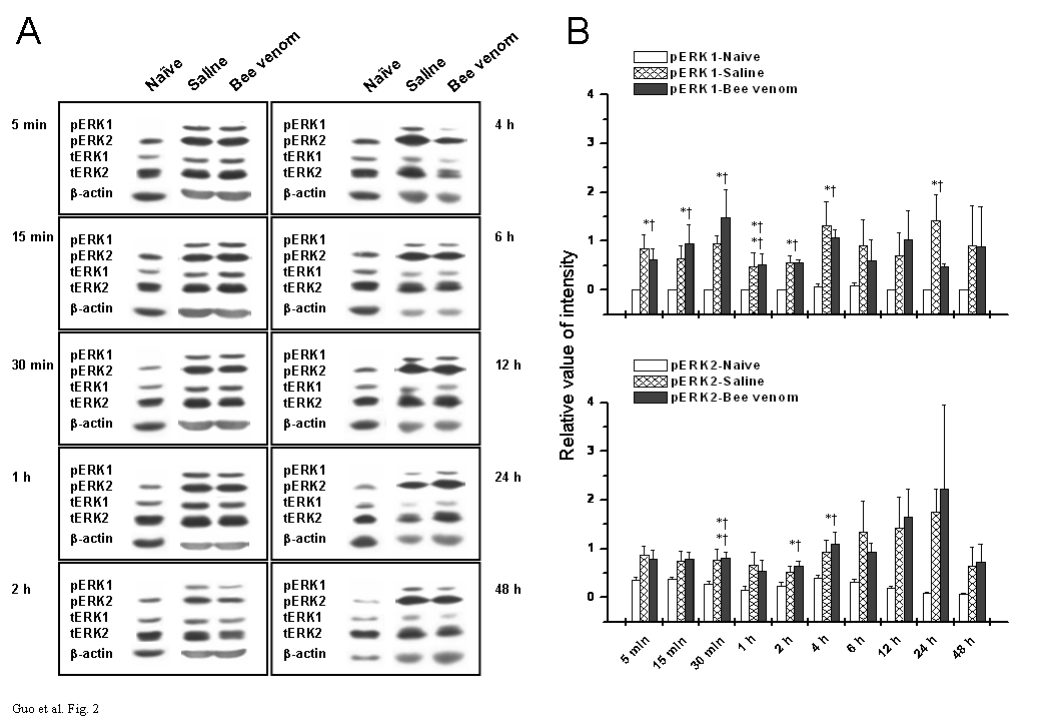
Temporal changes in phosphorylation and expression of ERKs in contralateral S1 area under three different states. (A) Representative Western blots of neocortical homogenates from three groups of rats at indicated time points. Left panel in each rectangle represents immunodetection of phosphorylated-ERK1/2 (pERK1/2), total ERK1/2 (tERK1/2), and beta-actin, which is included as loading control. Right panel in each rectangle shows raw bands corresponding to the left panel. (B) Densitometric quantification from (A) is shown. The pERKs bands were densitized and normalized to tERKs immunoblotted from the same membrane to demonstrate the phosphorylation levels of ERKs. Upper panel of B denotes the time course of ERK1 activation under three assigned states, while lower panel of B illustrates the time-related changes in activation of ERK2. Points represent the mean ± S.E.M. from three separate experiments. * *P *< 0.05, ** *P *< 0.01, saline-treated rats versus naïve control. † *P *< 0.05, †† *P *< 0.01, bee venom-treated rats versus naïve control.

Despite the reported prevalence and importance of SI area in pain sensory information processing [6-10], little attention has been paid to the molecular events occurring in this cortical area triggered by pain-evoking stimuli. Our study provided an initial examination about the differential expression profiles of ERKs in SI area under normal and different pain states. As shown in Fig. 2A, dramatic differences were observed in the immunoreactivity of tERK1 and tERK2 in contralateral S1 area from naïve rats, with ERK2 expressed more prominently than ERK1. Taken the results of spinal cord and SI area together, it is not difficult to find a reversion of ERKs expression mode in these two areas. With respect to the activated fraction of ERKs, pERK1 was rarely seen in SI area of naïve rats (Fig. 2A), however, pERK2 was normally expressed with a high level in this area at all time points we examined, although little differences occurred in the exact amounts of pERK2 among some time points.

In an attempt to unmask the state-dependent changes in the phosphorylation and total expression of ERK1 and ERK2 and hence illustrate the potential influences of pain-related behavioral consequence on ERK-mediated intracellular signaling pathways, we tested the temporal alterations in both pERK1/2 and tERK1/2 after s.c. saline or bee venom injection. Our immunoblotting results revealed that pERK1 was induced to express at a highly detectable level in contralateral SI area following both injection, when compared to the naïve control state (Fig. 2A, Table 1). In clear contrast, pain-induced elevation of pERK2 level was not so much evident as pERK1 when compared to its corresponding normal state, perhaps due to its high basal expression level in naïve rats (Fig. 2A, Table 2). A quantitative analysis of the data (Fig. 2B) further confirmed this phenomenon. We can see, from this histogram, that ERK1 was phosphorylated at almost every time point examined except for 6 h, 12 h, and 48 h, whereas ERK2 was activated at much less time points. No statistically significant differences were obtained between two groups of pain-experiencing rats (Fig. 2). In addition, total ERKs were unaltered by noxious stimulation given in our experiment (data not shown).

In addition, the ipsilateral side of SI area was removed from three groups of rats and SDS-solubilized tissue samples were subjected to Western blot analysis concomitantly. Almost the same increasing tendency was observed, but these changes have a more delayed and restricted temporal profile when compared to the contralateral alterations (Tables 1 and 2).

### Effects of s.c. injection of saline or bee venom on the phosphorylation of ERK1 and ERK2 in the hippocampus

**Figure 3:**
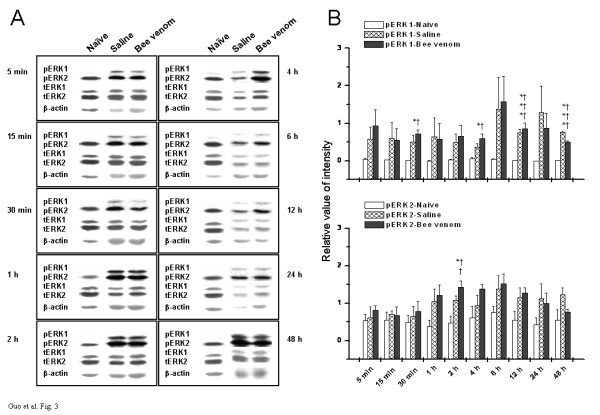
Temporal changes in phosphorylation and expression of ERKs in ipsilateral hippocampus under three different states. (A) Representative Western blots of hippocampal homogenates from three groups of rats at indicated time points. Left panel in each rectangle represents immunodetection of phosphorylated-ERK1/2 (pERK1/2), total ERK1/2 (tERK1/2), and beta-actin, which is included as loading control. Right panel in each rectangle shows raw bands corresponding to the left panel. (B) Densitometric quantification from (A) is shown. The pERKs bands were densitized and normalized to tERKs immunoblotted from the same membrane to demonstrate the phosphorylation levels of ERKs. Upper panel of B denotes the time course of ERK1 activation under three assigned states, while lower panel of B illustrates the time-related changes in activation of ERK2. Points represent the mean ± S.E.M. from three separate experiments. * *P *< 0.05, *** *P *< 0.001, saline-treated rats versus naïve control. † *P *< 0.05, †† *P *< 0.01, ††† *P *< 0.001, bee venom-treated rats versus naïve control.

Recently, evidence is accumulating that the hippocampal formation, an integral component of the "limbic" system [11,12], plays an important role in the cognitive-evaluative and affective-motivational components of pain experience [13,14]. Nevertheless, the definite role of phosphorylated ERKs in the hippocampal nociceptive regulation is not fully characterized yet. In our current study, rats were treated identically as described above, and then the time course study for pERK1 and pERK2 under three assigned status was conducted in the bilateral hippocampus. The results in Fig. 3A were representative of ipsilateral hippocampal determinations from analyses of three rats per group per time point. Almost the same type of isoform-dependent disparities in ERKs basal expression profile was observed from the raw immunoblotting bands. That is, paralleled with SI area but contrast to spinal cord (Fig. 1A and Fig. 2A), tERK2 showed much higher immunolabeling than tERK1, in spite of the fact that this difference between tERK1 and tERK2 was slightly smaller than that in SI area of cortex (Fig. 3A). Similarly, pERK2, but not pERK1, was normally detectable in the hippocampus from naive rats. It also seemed that pERK2 level in the normal hippocampus was a bit higher than that in normal SI area of cortex (Fig. 2A and Fig. 3A). Additionally, s.c. injection of either saline or bee venom significantly augmented the activation of ERK1 and ERK2 (Fig. 3A, Tables 1 and 2). Summarized data shown in Fig. 3B indicated that pERK2 was less changed by noxious stimulation compared with pERK1. Activation of ERK1 began to increase within 30 min of s.c. injection and remained at a relatively high level untill the end of the experimental period. However, statistically significant enhancement of pERK2 signal was only reached at 2 h after intraplantar treatment (Fig. 3B). At each time point showing enhanced phosphorylation of ERKs by pain stimulation, no significant differences were found between the transient pain and the persistent pain group (Fig. 3). There were no significant changes in total expression levels of ERK1 or ERK2 in ipsilateral hippocampus at any time point examined (data not shown).

Western blot analysis was also performed on tissue samples extracted from contralateral hippocampus of three groups of rats. The basal expression pattern for ERK1 and ERK2 was nearly identical to the ipsilateral side (data not shown). Both saline and bee venom injection resulted in a significant activation of contralateral hippocampal ERK1 at the early and late phase of the observation period (Table 1), whereas no significant changes were detected in phosphorylation of hippocampal ERK2 on the side contralateral to treatment (Table 2).

## Discussion

As a first step toward better understanding about the potential role of ERKs-mediated signaling pathways in central neuronal function under different states, we undertook a detailed analysis of spatiotemporally dynamic changes in phosphorylation (activation) and protein expression of two major ERK isoforms in the spinal cord dorsal horn, SI area and hippocampus under normal, physilogical pain (transient pain) and pathological pain (persistent pain) states. First, the present study showed that different ERK isoforms display different region-related expression profiles in the rat CNS under normal state. In general, ERKs are abundantly and ubiquitously expressed throughout the rat CNS and are reported to be present in the cerebral cortex, hippocampus, brain stem nuclei, cerebellum, thalamus and also in the spinal cord [44-47]. In our experiment, we detected certain amounts of ERK1 and ERK2 in normal spinal cord, SI area of cortex, and hippocampus using Western blot technique. This distributed expression profile of ERK1 and ERK2 was consistent with a previous study [44], which also found a wide distribution of ERK1 and ERK2 in all rat tissues examined, with the greatest amounts in brain and spinal cord, using antisera 956 and 837 derived from the C-terminal peptide predicted by the ERK1 cDNA, respectively. In spite of this global diffusion, regional differences in the relative abundance of ERK1 and ERK2 have also been described by previous reports [45,47]. Oritz and his colleagues found that, while ERK immunoreactivity was widely distributed in the rat brain, the relative abundance of ERK1 and ERK2 differed by close to 10-fold among the regions studied. In fact, they found that the ratio of ERK1 protein to ERK2 protein varied along a rostral-caudal gradient from a lowest of 0.16 in frontal cortex to a highest of 1.5 in pons/medulla. Moreover, this ratio in the spinal cord was even higher than that in pons/medulla [47]. Our blot immunolabeling of ERKs also presented a robust contrast between ERK1 and ERK2 immunoreactive intensity in the spinal cord dorsal horn, SI area and hippocampus. Interestingly, the relative differences between ERK1 and ERK2 immunoreactivity were quite the same as that previous study (see the present results). This point is further underscored by the Northern blotting data of Boulton et al. [45], who measured levels of ERK1 and ERK2 mRNA in gross subsections of brain. They found, consistent with our immunoblotting data, that ERK2 mRNA did show a rostral-caudal gradient in expression in different brain regions. However, what causes this kind of regional difference and what is the functional significance of this phenomenon remain largely unclear and await further elucidation.

The present study also provides information concerning the differential distribution of activated forms of ERKs, pERK1 and pERK2, in three examined areas in naïve rats. Obviously, ERK1 and ERK2 were rarely activated in the normal spinal cord (Fig. 1), but pERK2 was constitutively expressed with a high level in the SI area and hippocampus, although pERK1 was still hardly seen in these higher brain regions at the same time (Figs. 2 and 3). The reasons for this disparity in the distribution of activated ERK1 and ERK2 in different areas are not fully understood. A theoretically reasonable explanation might be attributed to different levels of ERK kinase, which catalyzes specifically the phosphorylation of ERKs at both a threonine and a neighboring tyrosine residue and activates them [48-50]. Oritz and his colleagues had also investigated the regional distribution of ERK kinase using blot immunolabeling procedures and shown that the highest levels of ERK kinase immunoreactivity were present in nucleus accumbens, hippocampus, substantia nigra, and caudate/putamen, with the lowest levels observed in cerebellum and pons/medulla. They also pointed out that the spinal cord contained still lower levels of ERK kinase immunoreactivity [47]. Hence, combined with our findings, we extrapolate from the previous results that it is probably due to the differential distribution of ERK kinase, MEK1 or MEK2 or other unknown additional members of this kinase family, that leads to the differential distribution of pERK1 and pERK2 between spinal and supraspinal level under normal state observed in the present study. Of course, this presumption still needs additional verification in future studies.

In the present experiments, we also addressed whether or, if so, to what extent peripherally-induced transient or persistent pain state will influence ERKs phosphorylation status and their distribution patterns in the spinal cord and high-level brain structures. We show here regional selectivity in the phosphorylation of ERK isoforms following peripheral noxious stimulation, with different sensitivity and responsibility between ERK1 and ERK2 found in different areas. In the spinal cord dorsal horn, both pERK1 and pERK2 were dramatically elevated in response to transient or persistent pain stimulation (Fig. 1, Tables 1 and 2). However, the ERK2 enzyme seems to be more sensitive and exhibit stronger responses than ERK1 enzyme. In SI area or hippocampus, in contrast, pERK2 was less changed or changed not so evident and remarkable as pERK1 (Figs. 2 and 3, Tables 1 and 2). These differential activation properties of ERK1 and ERK2 are in large accordance with previous reports [51,52]. Several factors may contribute to this kind of ERKs activation during pain state, such as glutamatergic receptors (NMDA receptor [53], AMPA receptor [54], or metabotropic glutamate receptor [55]), growth factors (nerve growth factor [56] or brain-derived neurotrophic factor [57]), and so on. However, the reasons for these differential activation patterns of different isoforms are not clear. We suggest, to some extent, that it maybe due to the differential activation and regulation of upstream activators for ERK1 and ERK2 (MEK1 or MEK2, for example) in different areas under the pain states. A substantial body of evidence from brain imaging, lesion and electrophysiological studies indicates that different components of the nociceptive system may be preferentially involved in different aspects of the complex experience of pain [4-8]. Combined differential participation of the spinal cord, SI area, and hippocampus in the multidimensional aspects of pain experience with region- and isoform-dependent responses of pERK1 and pERK2 to peripheral noxious stimulation observed in the present study, we propose a hypothesis that this differential activation of ERK1 and ERK2 across distinct regions under pain state might suggest the possibility for a different function of specific ERK members in the nociceptive processing and further provide a molecular basis for the differential involvement of individual components of nociceptive system in the diverse features of pain.

It has been well documented that pathological pain (persistent or chronic pain) differs greatly from physiological pain (transient pain) in regards of etiology, symptom, mechanisms and pathogenesis [33-36]. Therefore, a difference in the intracellular signaling mechanisms of these two pain states might also be expected. In the present study, using ERK as a marker, we sought to address this issue by virtue of two well-developed animal models: s.c. injection of 0.9% isotonic saline solution as the transient pain model, and s.c. injection of whole bee venom solution as the persistent pain model. In our pilot experiments, we did not observe marked paw flinching (or lifting) responses behaviorally (paralleled with previous observations [37,58]), nor did we find long-lasting increase in spontaneous spike discharges of spinal cord dorsal horn neurons electrophysiologically following intraplantar saline injection in conscious rats (data not shown). However, saline-treated rats did exhibit typical behavioral manifestation of acute, localized, transient pain during the process of injection, such as slight withdrawal of the hindpaw, the desire to escape, and even vocalization some times. All of these observations, therefore, led us to the conclusion that s.c. injection of isotonic 0.9% saline can indeed elicit transient, but not persistent pain in conscious rats. Somewhat unexpectedly, our present immunoblotting results did not reveal any significant differences in the activation of ERK1 or ERK2 between saline- and bee venom-treated rats with regards to either response intensity or duration. This result is in contrast to a previous study [59], which showed a significant increase of pERK in the spinal cord and hippocampus following intrathecal (i.t.) substance P injection, another well-characterized pain model [60], but not after i.t. saline treatment. This discrepancy between their results and ours may be ascribed to many differences in experimental design and procedure, such as the animal species used (rat vs. mouse) or the route of drug administration (s.c. vs. i.t.) or the observation period (long-term vs. short term). Consistent with our present findings, Galan et al. [29] has also reported that intraplantar saline injection resulted in a 2.5-fold activation of spinal ERK in juvenile rats, but this activation only persisted until 45 min after intraplantar injection. Our results in the current study extend their findings by showing an even longer activation of ERKs to 24–48 h after intraplantar treatment with saline in both spinal cord and higher-level brain structures. Another new finding of this study, in comparison with that previous report, was that we observed differential response patterns between different ERK isoforms in response to peripherally-evoked pain state. The precise mechanisms for this saline-induced phosphorylation of ERKs are not clear. However, since several intracellular kinase cascades converge on MAPK activation [23,61-63], it is not unreasonable to speculate that ERK, as a member of highly conserved and ubiquitously distributed MAPK family [15-17], might serve as a constitutive integrator of multiple inputs from extracellular environment, so that even transient pain could immediately activate it and hence trigger a series of adaptive changes in the intracellular signal transduction.

Another most important point to be noted is that the isotonic and sterile saline solution we used in the present study has a low PH value (PH = 6.4). There is ample evidence demonstrating that acidic saline treatment, intramuscular injection or topical application, can produce a bilateral, long-term mechanical hyperalgesia in rats [64,65]. Thus, the present results could not exclude the possibility that the long-lasting enhanced activation of ERKs by saline-induced transient pain is partially attributable to the effects exerted by its low PH nature. Besides, one of the more interesting and striking findings from the present study is that unilateral injection of saline or bee venom solution elicited bilateral phosphorylation of ERKs in three areas examined, although the effects of the other side were not so much evident and usually had a more delayed and restricted temporal profile (see Tables 1 and 2). More recently, different experiments employing multidisciplinary approaches in animals and humans have clearly demonstrated that the bee venom treatment could produce not only persistent spontaneous nociception associated with long-term primary mechanical and heat hyperalgesia at injection site, but also heat hyperalgesia identified in the surrounding secondary area and even the remote contralateral non-injection limb, the so-called "mirror-image" thermal hyperalgesia [33,38-40,66-69]. Our observations concerning the bilateral activation of ERKs following bee venom injection were highly correlated with the above reports. According to our previous investigations, it is further suggested that a communication mediated by commissural interneurons in the rat CNS might contribute to the above-mentioned bilateral ERKs activation as well as mirror-image hyperalgesia identified in the bee venom model [66,70]. With respect to the saline-induced bilateral ERKs activation, we have reason to believe that the low PH nature of the saline solution might be responsible, at least in part, for this phenomenon [64,65].

The present study also tested the time course of this noxious stimulation-induced ERKs activation, from 5 min to 48 h. The temporal pattern (lasting 24–48 h) of bee venom-induced spinal or cortical ERKs activation differed substantially from the transient (<2 h) ERKs activation reported after intradermal injection of capsaicin or formalin [26,27,51], but is mostly in accordance with the ERKs activation induced by intraplantar Complete Freund's adjuvant (CFA) administration [30]. This long-term activation of ERKs correlates well with our previous behavioral, morphological and electrophysiological studies [33,38-43,71]. On the basis of these findings, therefore, we suppose that ERKs activation in the early phase of bee venom-evoked inflammation, from 5 min to 1 h, might be involved in the development or maintenance of persistent spontaneous nociception (lasting 1–2 h); while the later phase of ERKs activation, from 2 h to 48 h, might participate in the bee venom-produced thermal or mechanical hyperalgesia (lasting 24–72 h). This point is further confirmed by our previous pharmacological study [72], in which i.t. administration of U0126, a widely used specific MEK inhibitor [73], significantly blocked the induction and maintenance of melittin-induced persistent spontaneous nociception and heat hyperalgesia, while the same treatment only significantly suppressed the induction, but not maintenance, of melittin-induced mechanical hypersensitivity. Our recent studies have demonstrated that melittin, which composes over 50% of the whole bee venom [74], is the major algogenic component for the bee venom-produced long-term changes in peripheral and central neural plasticity as well as abnormal pain behaviors [39,75], Therefore, these spinal pharmcological results and our present blot immunolabeling data add to an incrementing body of evidence for the functional involvement of phosphorylated ERKs in mediating nociceptive signal transmission and sensitization in the bee venom model.

## Conclusion

In the present study, we provided a new line of evidence showing region- or state-related differences in expression and activation between ERK1 and ERK2 along the pain-related CNS pathways in rats. Under normal state, the relative abundance of constitutive ERK1 and ERK2 differs greatly among specific regions in the rat CNS, while under pain state, both ERK1 and ERK2 can be effectively phosphorylated with a long-term duration by both transient and persistent pain, but with their response patterns being different from each other across distinct regions as well. The long-lasting ERKs activation induced by peripheral bee venom injection is highly correlated with our previous behavioral, electrophysiological, morphological and pharmacological observations, lending further support to the functional importance of ERKs-mediated signaling pathways in the processing of negative consequence of pain associated with sensory, emotional and cognitive dimensions.

## Methods

### Animal model

All experiments were carried out on male Sprague-Dawley rats (Animal Center of Capital Medical University, Beijing, PR China) weighing from 220 to 250 g. The animals were housed five per cage with food and water available *ad libitum*, and kept under controlled conditions of temperature (20–25°C) and light (12 h light/12 h dark cycle). This animal protocol was approved by University Institutional Animal Care and Use Committee of Capital Medical University and was consistent with the ethical guidelines of the International Association for the Study of Pain for pain research in conscious animals [76]. All efforts were made to minimize animal suffering and to reduce the number of animals used. The rats were randomly divided into three groups: (1) rats without any treatment as naïve control (normal state, n = 3/time point); (2) rats with s.c. injection of 0.9% sterile, isotonic saline solution (PH = 6.4) as the physiological pain model (transient pain state, n = 3/time point); (3) rats with s.c. injection of whole bee venom solution as the pathological pain model (persistent pain state, n = 3/time point).

### Administration of drugs

Bee venom was lyophilized whole venom of *Apis mellifera *(Sigma, St. Louis, MO) dissolved in 0.9% sterile saline. A volume of 50 μl saline containing 0.1 mg bee venom was used during the whole experiment, because it was shown that 0.1–0.2 mg/50 μl was the optimal dose to produce a prolonged pain-related behavioral response [37]. Another parallel group of rats received an equal volume of 0.9% isotonic, sterile saline solution (PH = 6.4, Sodium Chloride Injection for medical use, purchased from Yangzhou Zhongbao Pharmaceutical Co., Ltd.). Subcutaneous injection of bee venom or saline was administered into the posterior plantar surface of the right hind paw of rats as reported previously [38]. Briefly, a 26-gauge 5/8 inch needle connected to a 0.1 ml syringe was used and the solution was delivered as rapidly as possible while the animal was immobilized.

### Tissue processing and sample preparation

In order to examine the spatial and temporal patterns of ERK1/2 phosphorylation and expression, all three groups of rats were anesthetized with intraperitoneal injection of sodium pentobarbital (50 mg/kg, i.p.) and decapitated at various time points (5 min, 15 min, 30 min, 1 h, 2 h, 4 h, 6 h, 24 h, 48 h) after the injection. The bilateral SI area and hippocampus tissue were extracted sequentially according to the atlas of Paxinos and Watson [77] and then placed in an ice-cooled glass dish. Subsequently, the whole spinal cord of each rat was removed by pressure expulsion with saline into the same dish. The lumborsacral enlargement was identified as required, excised into two sections, ipsilateral and contralateral to the injected side, from which the dorsal horn part was eventually dissected. At last, all of these sections were labeled and snap-frozen in liquid nitrogen (-80°C) until further treatment.

The sectioned tissues were homogenized at 4°C in homogenizing buffer [50 mM Tris-Cl, pH 7.5, containing 2 mM dithiothreitol, 2 mM EDTA, 2 mM EGTA, 50 μM (2-aminoethyl)-benzenesulfonylfluoride hydrochloride, 5 mg/ml each of leupeptin, aprotinin, pepstatin A, and chymostatin, 50 mM KF, 50 nM okadaic acid, 5 mM sodium pyrophosphate, and 2% SDS] and sonicated to dissolve the tissue completely. Then, the protein amounts of samples were determined by BCA kit (Pierce, USA).

### SDS-PAGE and immunoblotting

The levels of phosphorylation and protein expression of ERK1/2 were determined using Western blot technique as described previously [78]. Briefly, 30 μg of protein from the tissue homogenates of each sample was loaded onto 10% SDS-PAGE gel. Then, the protein was separated by ice-cooled electrophoresis, after which separated protein was electrophoretically transferred onto nitrocellulose membranes (Schleicher & Schuell, USA) at 4°C. The transferred membrane was firstly blocked by incubating in blocking buffer (10% non fat dry milk in Tris-buffered saline containing 0.05% Tween 20) for 1 h at room temperature (RT).

After washing 3 × 10 min in TTBS (20 mM Tris-Cl, pH 7.5, containing 0.15 M NaCl and 0.05% Tween 20), the membranes were incubated with following primary antibodies at a 1:1000 dilution for 4 h at RT: rabbit anti-mouse phospho-p44/p42 ERK1/2 pAb (Cell Signaling Technology, USA); rabbit anti-mouse pan-p44/p42 ERK1/2 pAb (Cell Signaling Technology, USA); or mouse anti-human beta-actin mAb (Sigma, USA). The membranes were extensively washed three times with TTBS and then incubated with horseradish peroxidase-conjugated goat anti-rabbit or goat anti-mouse secondary antibody (Bio-Rad, USA) at a 1:3000 dilution for 2 h at RT. After washing another three times with TTBS, immunoblotting signals were visualized by treatment with ECL reagent (PerkinElmer Life Sciences, USA) and exposure to film.

### Quantitative analysis and data presentation

Densitometric quantification of immunoreactive bands was performed with GelDoc-2000 Imagine System (Bio-Rad, USA). Since the primary antibodies we used in the present experiment detected both ERK1 (p44) and ERK2 (p42), which have different molecular weights, two bands (44 kDa and 42 kDa) were visualized at last. So both of the bands were captured with the image analysis system and the intensity of each band was quantified separately. The value of the relative optical density of each band corresponding to phospho-ERK1/2 was normalized to the value of total-ERK1/2 to demonstrate the phosphorylation level; the relative density of each band corresponding to total-ERK1/2 was normalized to the value of beta-actin band to illustrate the protein expression level. All presented data were expressed as means ± S.E.M. from three independent experiments.

### Statistical analysis

Statistical analysis was conducted by one way analysis of variance (ANOVA) followed by individual post-hoc multiple comparisons (Fisher's PLSD test). A statistical difference was accepted as significant at P < 0.05.

## Authors' contributions

S-WG and M-GL conducted the majority of the immunoblotting studies. M-GL, Y-LL performed some important part of the experiments and assisted in the writing of the manuscript. L-YR, Z-ML, H-YY, J-FH, HL quantitated many of the films using image analysis. L-FZ, C-YG, X-YC, Y-YA and JL played roles in technical assistance and establishment of the methods. JC performed design of the study, supervision of the experiments, analysis of the data and composing and proofing the manuscript. All authors listed have read and approved the whole manuscript.
